# Serum HER2 extra-cellular domain, S100ß and CA 15-3 levels are independent prognostic factors in metastatic breast cancer patients

**DOI:** 10.1186/s12885-016-2448-1

**Published:** 2016-07-07

**Authors:** Amélie Darlix, Pierre-Jean Lamy, Evelyne Lopez-Crapez, Antoine Laurent Braccini, Nelly Firmin, Gilles Romieu, Simon Thezenas, William Jacot

**Affiliations:** Department of Medical Oncology, Institut régional du Cancer de Montpellier, 208 rue des apothicaires, 34298 Montpellier, France; Department of Clinical Research, Clinique Beausoleil, 19 Avenue de Lodève, 34070 Montpellier, France; Department of Biology and Oncogenetic, Institut régional du Cancer de Montpellier, 208 rue des apothicaires, 34298 Montpellier, France; Translational Research Unit, Institut régional du Cancer de Montpellier, 208 rue des apothicaires, 34298 Montpellier, France; Centre Azuréen de Cancérologie, 1 place du Docteur Jean Luc Broquerie, 06250 Mougins, France; Biometrics unit, Institut régional du Cancer de Montpellier, 208 rue des apothicaires, 34298 Montpellier, France

**Keywords:** Breast cancer, Metastases, Tumor markers, Tumor heterogeneity, Serum HER2, S100ß, Neuron-specific enolase, Matrix metalloproteinase 9

## Abstract

**Background:**

Metastatic breast cancer (MBC) prognosis is highly variable, depending on various factors such as the biological subtype, the performance status, disease extension…. A better evaluation of a patient’s prognostic factors could allow for a more accurate choice of treatments. The role of serum tumor markers remains, however, unclear in this population. Considering the recent interest in phenotypic changes and tumor heterogeneity during breast cancer progression, additional tumor markers could be interesting in this setting.

**Methods:**

Two hundred fifty MBC patients treated at the Montpellier Cancer Institute (2008–2015) were retrospectively selected, based on the availability of frozen serum samples. The usual MBC clinical and pathological variables were collected, altogether with Cancer Antigen 15-3 (CA15-3), Carcinoembryonic Antigen (CEA), HER2 extra-cellular domain (ECD), Neuron Specific Enolase (NSE), S100ß protein and Matrix Metalloproteinase 9 (MMP-9) serum levels in order to determine their prognostic value.

**Results:**

With a median follow-up of 40.8 months, median overall survival was 16.2 months (95 % CI 12.4–20.6). In multivariate analysis, the performance status, brain or subcutaneous metastases, the number of previous metastatic chemotherapy lines and the tumor biological subtype were independent prognostic factors. Elevated CA 15-3 (HR = 1.95, IC 95 % 1.31–2.93, *p* = 0.001), HER2 ECD (regardless of tumor HER2 status, HR = 2.51, IC 95 % 1.53–4.12, *p* < 0.001) and S100ß (HR = 1.93, IC 95 % 1.05–3.54, *p* = 0.033) serum levels were independently associated with a poor outcome.

**Conclusions:**

Serum CA 15-3, HER2 ECD and S100ß could represent useful independent prognostic factors in MBC. Of particular interest is the independent value of serum HER2 ECD levels, regardless of the tumor HER2 status, possibly linked to metastatic tumor heterogeneity.

## Background

The survival of patients with metastatic breast cancer (MBC) has improved over the past decades with the use of new therapeutic agents [1]. However, the outcome remains poor (median overall survival [OS] of 21–30 months) [1, 2]. MBC prognosis is highly variable depending on various factors, with survivals ranging from a few months to decades. This variability probably reflects the biological heterogeneity among MBC [3]. Indeed, in a study on 1 038 MBC patients, the median OS was 27 months for tumors with overexpression of hormone receptors (HR) (HR-positive tumors) compared with 9 months for HR-negative tumors [4]. Likewise, the median OS for HER2 (Human epidermal growth factor receptor 2)-positive/HR-positive tumors and triple negative tumors were 34.4 and 8.8 months, respectively, in a series of 815 MBC patients [5]. Given this important variability in terms of outcomes, a better evaluation of a given patient’s prognostic factors could allow for a more accurate choice of the therapeutic strategy.

To date, several clinical and biological prognostic factors have been reported, including patients’ characteristics (age and performance status), previous medical history (prior chemotherapy [CT] for MBC treatment), disease extension (number and location of metastatic sites) and tumor biology. A high number of metastatic sites, the presence of visceral metastases compared to bone and/or soft tissue metastases only, and the presence of liver or brain metastases (BM) are clinical features associated with a poor outcome in various studies [4–6]. Tumor biology remains a cornerstone of patients’ prognosis, linked to prognostic and predictive factors (HR and HER2 expression). HR-positive tumors have been associated with a better outcome, and with response to hormone therapy [4, 7, 8]. Regarding the HER2 status, series from the pre-Trastuzumab era have demonstrated a negative prognostic value of the HER2 amplification in MBC [9]. Since the introduction of Trastuzumab, however, this effect has been reversed and HER2 amplification has been associated with a better outcome in recent series [5, 10]. In the series published by Dawood et al., patients with HER2-positive MBC receiving Trastuzumab had a better outcome than patients with HER2-positive MBC not receiving Trastuzumab, or than patients with HER2-negative MBC [10]. Overall, the different biological subtypes according to the HR and HER2 statuses have been correlated with variable prognosis in MBC as well as in early breast cancer (EBC) [5]. High Lactate Dehydrogenase (LDH) and low albumin serum levels have also been reported as poor prognostic factors in MBC in several studies [11–13].

The role of serum tumor markers in predicting outcome remains, however, unclear in patients with MBC. High Cancer Antigen 15-3 (CA 15-3) and Carcinoembryonic Antigen (CEA) serum levels seem to be associated with a poorer survival [14–16]. The prognostic value of serum HER2 extra-cellular domain (ECD) has been shown in HER2-positive MBC patients, high serum HER2 ECD levels being associated with a poor outcome [17, 18]. Few studies have investigated this question in HER-negative tumors: some authors have suggested a prognostic value of the serum HER2 ECD in HER2-negative tumors as well [19].

Other biomarkers have been investigated to predict outcome in MBC patients, including circulating tumor cells and growth factors [14, 18]. Considering the recent interest in phenotypic changes and tumor heterogeneity during breast cancer progression, it could be interesting to evaluate additional tumor markers in this setting to better assess tumor heterogeneity in a non-invasive manner.

Among these biomarkers, the Neuron Specific Enolase (NSE), a neuronal marker, seems a good candidate. An elevated serum NSE level in MBC could reflect either a neuroendocrine differentiation of the tumor or a central nervous system extent of the disease, and be linked with poor outcome. Serum NSE has been studied in small-cell lung cancers, and in various neurological conditions such as stroke or Creutzfeld-Jakob disease [20–22]. However, to our knowledge, its prognostic value has never been investigated in MBC.

The Matrix Metalloproteinase 9 (MMP-9), a proteolytic enzyme that degrades the extra-cellular matrix components, is involved in tumor invasion and metastatic dissemination [23]. Its serum level has been associated with several solid tumors including breast cancer [24–26]. In breast cancer, it has been correlated with poor prognostic in one study only [25]. Moreover, a recent study on gastric cancer has reported interactions between MMP-9 and HER2 in invasion processes [27].

The serum S100ß protein is involved in cell proliferation [28]. Its prognostic value has been demonstrated in melanoma [29]. Being an astrocytic marker, its elevation in serum is a marker for an alteration of the blood-brain barrier. Therefore, serum S100ß has been widely studied in various neurological diseases [21, 30]. Due to the high frequency of asymptomatic central nervous system involvement in breast cancer, serum S100ß could have a prognostic value and be an interesting serum biomarker in MBC [31].

The aim of the present study was to evaluate the prognostic value of serum tumor markers, namely CA15-3, CEA, HER2 ECD, NSE, MMP-9 and S100ß in a series of MBC patients. We hypothesized that these biomarkers serum levels were significantly associated with OS.

## Methods

### Design

We conducted a retrospective, monocentric study on MBC patients treated at the Montpellier Cancer Institute between 2008 and 2015.

### Patients

MBC patients were retrospectively identified for the purposes of a case-control study conducted by our team in MBC patients with BM (submitted), by reviewing the medical records of the MBC patients from the Montpellier Cancer Institute database between 2008 and 2015. Inclusion criteria were: patient ≥ 18 years old; histologically-confirmed MBC; availability of the HR and HER2 statuses of the primary tumor; availability of a frozen serum sample performed at the metastatic phase, for biomarker determination. Patients with history of other cancer(s) were excluded.

### Objectives

The primary objective was to evaluate the prognostic value of six serum biomarkers (namely, CA 15-3, CEA, HER2 ECD, NSE, MMP-9 and S100ß) in patients with MBC. The secondary objectives were to evaluate the prognostic value of other usual clinical and biological prognostic factors.

### Clinical and biological data

Clinical and biological data were collected by reviewing the medical records of the selected patients: demographical, clinical (date of diagnosis of breast cancer and metastatic disease; metastatic status at diagnosis; inflammatory breast cancer; treatment history), and biological data (histological grade of the primary tumor, HR and HER2 statuses). The tumor was considered HR-positive when more than 10 % of cells were labeled in immunohistochemistry (IHC) or when the concentrations of estrogen (ER) and progesterone receptors (PR) using the radio ligand binding method were above 10 ng/mL and 50 ng/mL, respectively. The tumor was considered HER2-positive if the primary tumor was scored 3+ by IHC or if the HER2 gene was amplified by fluorescence or chromogenic in situ hybridization (FISH/CISH) for IHC 2+ cases. For cases with HR and/or HER2 statuses changes over time, the biology used was that of the most recent sample. For cases of synchronous or asynchronous bilateral cancer with discrepant HR and/or HER statuses, the most unfavorable biology was used: higher histological grade, HR-negative, HER2-negative (Trastuzumab era).

### Analysis of serum biomarkers

The selected serum samples were extracted from the Biological Resource Center of the Montpellier Cancer Institute (Biobank number BB-0033-00059) (samples processed within one hour after sampling and stored at −80 °C in serum aliquots). The biologists performing the analyses were blinded to the study endpoints. HER2 ECD, NSE and MMP-9 serum levels were measured by ELISA using commercially available ELISA assays (Human MMP-9 Quantikine Kit R&D Systems, Minneapolis, MN, USA for MMP-9, ELSA-NSE RIA kit, Cisbio assays, Gif sur Yvette, France for NSE, and Nuclea Diagnostic Laboratories kit, LLC for HER2 ECD) according to the manufacturer’s instructions. For MMP-9, a duplicate analysis was performed, but no duplicate analyses were deemed necessary. S100ß serum levels were measured with the Elecsys S100 Immunoassay (Roche Diagnostics GmbH, Mannheim, Germany). Other biological parameters, including CA 15-3 (ELSA-CA15-3 Cisbio assays Gif sur Yvette France) and CEA (Elecsys CEA test Roche Diagnostics, Meylan, France), had previously been analyzed for clinical purposes and were collected. As we evaluated the prognostic value of biomarkers, the manuscript adheres to the REMARK guidelines.

### Statistical analysis

Categorical variables were reported: number of missing data, number and percentage for each variable modality. For continuous variables, number of missing data, mean, standard deviation, median and range values were computed. OS delay was measured from the date of the serum sample to the date of death from any cause. Patients alive without events were censored at the closing date of the study analysis (March 30^th^, 2015). The OS was estimated according to the Kaplan-Meier method, and presented as medians with their 95 % confidence intervals (95 % CIs) and survival rates (in percent, with 95 % CIs) [32]. The median duration of follow-up was estimated using a reverse Kaplan-Meier method and presented with its 95 % CIs. As there are no validated cut-offs in MBC patients, different cut-offs reported in previous studies were used to investigate the prognostic value of the serum HER2 ECD (cut-offs 15 ng/mL and 30 ng/mL [17, 33]), NSE (cut-off 12.5 μg/L [34]), MMP-9 (cut-off 50 ng/mL [35]) and S100ß (cut-offs 0.12 μg/L and 0.072 μg/L [33]). The following cut-offs were used for the usual breast cancer biomarkers: 30 U/mL for CA 15-3 and 10 ng/mL for CEA. To investigate prognostic factors, a multivariate analysis was performed using the Cox’s proportional hazards regression model with a stepwise procedure. Hazard ratios (HR) with 95 % CIs were calculated to display risk changes. All *p* values reported were two-sided, and the significance level was set at 5 % (*p* < 0.05). Statistical analysis was performed using the STATA 13 software (Stata Corporation, College Station, TX).

### Ethical considerations

This study was reviewed and approved by the Montpellier Cancer Institute Institutional Review Board (ID number ICM-URC-2014/73). Considering the retrospective, non-interventional nature of this study, no specific consent was deemed necessary by the clinical research review board of the Montpellier Cancer Institute.

## Results

### Patients’ characteristics

A total of 250 women with MBC were included in the study. The clinical and biological characteristics of patients at baseline are presented in Table 1. Median age at the time of the serum sample was 58.4 years old (range 26.4–87.2). The most represented histological subtype was ductal carcinoma in 82.9 % of cases. Tumor biology was distributed as follows: HER2+/HR+ in 23.2 %, HER2+/HR- in 24.4 %, HER2-/HR+ in 25.6 % and triple negative in 26.8 % of cases. Among the 250 patients, 70 presented with synchronous metastases at breast cancer first diagnosis. The median number of previous chemotherapy (CT) lines was 1 (range 0–9; no previous CT 24.4 %, one or two CT lines 45.2 %, and more than two CT lines 30.4 %). 40.4 % of patients had received a previous anti-HER2 treatment (83.2 % of patients with a HER2-positive tumor). The metastatic-free interval (MFI) was over 24 months for 55.6 % of patients. At the time of the serum sample, 60.8 % of patients had liver metastases, 56.8 % bone metastases, 53.6 % lymph nodes metastases, 50.0 % had lung metastases, 35.2 % of patients had BM, 22.0 % pleural metastases, 17.2 % had subcutaneous metastases and 43.6 % had metastases at other sites. A vast majority of patients had at least two metastatic sites (86.0 %). Only 13 patients (5.2 %) had only bone and/or subcutaneous metastases. Most patients had a good performance status (ECOG status ≤ 2 in 90.6 %). Albumin serum level was available for 158 (63.2 %) patients and was low in 23 cases (14.6 %). Serum LDH level was high in 43.3 % of the 90 cases with a reported value.

**Table 1:** Patients’ clinical and biological characteristics

Initial characteristics	
*Tumor biology group, n (%)*	
HER2+ HR+	58 (23.2)
HER2+ HR-	61 (24.4)
HER2- HR+	64 (25.6)
Triple negative	67 (26.8)
* ER status, n (%)*	
Negative	128 (51.2)
Positive	122 (48.8)
* PR status, n (%)*	
Negative	186 (74.7)
Positive	63 (25.3)
Missing	1
* HER2 status, n (%)*	
Negative	131 (52.4)
Positive	119 (47.6)
* Histological subtype, n (%)*	
Ductal carcinoma	203 (82.9)
Other subtypes^a^	42 (17.1)
Missing	5
* Histological grade (SBR), n (%)*	
1 or 2	103 (45.8)
3	122 (54.2)
Missing	25
* Inflammatory BC, n (%)*	
No	216 (90.8)
Yes	22 (9.2)
Missing	12
* Metastatic status at BC diagnosis, n (%)*	
M0	174 (71.3)
M1	70 (28.7)
Mx	6
Patients’ characteristics at the time of the serum sample	
* Median age in years (range)*	58.4 (26.4–87.2)
* Age group, n (%)*	
< 50	77 (30.8)
50 to 70	132 (52.8)
> 70	41 (16.4)
* Median number of lines of CT (range)*	1 (0–9)
* Number of lines of CT, n (%)*	
0 line	61 (24.4)
1 or 2 line(s)	113 (45.2)
> 2 lines	76 (30.4)
* Previous anti-HER2 treatment, n (%)*	
No	149 (59.6)
Yes	101 (40.4)
* Brain metastases, n (%)*	88 (35.2)
* Liver metastases, n (%)*	152 (60.8)
* Bone metastases, n (%)*	142 (56.8)
* Lung metastases, n (%)*	125 (50.0)
* Lymph node metastases, n (%)*	134 (53.6)
* Subcutaneous metastases, n (%)*	43 (17.2)
* Pleural metastases, n (%)*	55 (22.0)
* Metastases of other sites, n (%)*	109 (43.6)
* ECOG status, n (%)*	
0	70 (30.2)
1	106 (45.7)
2	34 (14.7)
3	22 (9.5)
Missing	18
* Anemia, n (%)*	
No	38 (25.9)
Yes	109 (74.1)
Missing	103
* Leucopenia, n (%)*	
No	118 (82.5)
Yes	25 (17.5)
Missing	107
* Neutropenia, n (%)*	
No	129 (89.0)
Yes	16 (11.0)
Missing	105
* Lymphopenia, n (%)*	
No	82 (59.4)
Yes	56 (40.6)
Missing	112
* Thrombopenia, n (%)*	
No	128 (88.3)
Yes	17 (11.7)
Missing	105
* Elevated LDH, n (%)*	
No	51 (56.7)
Yes	39 (43.3)
Missing	160
* Elevated serum CEA, n (%)*	
No	154 (66.4)
Yes	78 (33.6)
Missing	18
* Elevated serum CA 15-3, n (%)*	
No	92 (39.7)
Yes	140 (60.3)
Missing	18
* Elevated serum HER2 ECD (cut-off 15 ng/mL)*	
No	107 (42.8)
Yes	143 (57.2)
Missing	0
* Elevated serum HER2 ECD (cut-off 30 ng/mL)*	
No	187 (74.8)
Yes	63 (25.2)
Missing	0
* Elevated serum NSE (cut-off 12.5 μg/L)*	
No	76 (30.5)
Yes	173 (69.5)
Missing	1
* Elevated serum MMP9 (cut-off 50 ng/mL)*	
No	6 (2.4)
Yes	244 (97.6)
Missing	0
* Elevated serum S100ß (cut-off 0.12 μg/L)*	
No	224 (92.2)
Yes	19 (7.8)
Missing	7
* Elevated serum S100ß (cut-off 0.072 μg/L)*	
No	183 (75.3)
Yes	60 (24.7)
Missing	7
* Low protein level, n (%)*	
No	32 (74.4)
Yes	11 (25.6)
Missing	119
* Low albumin level, n (%)*	
No	135 (85.4)
Yes	23 (14.6)
Missing	92
Follow-up	
* Status as last follow-up, n (%)*	
Alive	77 (30.8)
Dead	173 (69.2)
* Cause of death, n (%)*	*(174 deceased)*
Oncological disease	153 (87.9)
Non-oncological	4 (2.3)
Toxic	4 (2.3)
Unknown	13 (7.5)

*Abbreviations*: *ER* estrogen-receptors, *PR* progesterone-receptors, *SBR* Scarf, Bloom and Richardson, *BC* breast cancer, *CT* chemotherapy, *LDH* Lactate Deshydrogenase, *CEA* Carcinoembryonic Antigen, *CA 15-3* Cancer Antigen 15-3, *HER2-ECD* HER2 extra-cellular domain, *NSE* Neuron Specific Enolase, *MMP-9* Matrix Metalloproteinase 9

^a^ lobular carcinoma (*n* = 21), mucinous carcinoma (*n* = 1), papillary carcinoma (*n* = 3), medullary carcinoma (*n* = 1), mixed ductal and lobular carcinoma (*n* = 12), other histological subtypes (*n* = 4)

### Serum CA 15-3, CEA, HER2 ECD, NSE, MMP-9 and S100ß

The median serum biomarker levels were: 38.0 U/mL (range 8.0–1988.0) for CA 15-3, 4.0 ng/mL (range 1.0–5122.0) for CEA, 13.6 ng/mL (range 2.8–280.0) for HER2 ECD, 9.0 μg/L (range 1.9–57.3) for NSE, 341.0 ng/mL (range 38.7–2051.0) for MMP-9 and 0.05 μg/L (range 0.0–0.6) for S100ß. The proportion of patients with high values is presented for each serum tumor marker in Table 1. Serum CA 15-3 and CEA were elevated in 60.3 % and 33.6 % of the 232 patients with known values. Serum HER2 ECD (cut-off 15 ng/mL) and MMP-9 (cut-off 50 ng/mL) were elevated in 42.8 % and 97.6 % of the 250 patients with known values, respectively. Serum NSE was high in 30.5 % of the 249 patients with known values, and serum S100ß (cut-off 0.12 μg/L) in 7.8 % of the 243 patients with known values. All but two patients had at least one elevated biomarker among S100ß, NSE, MMP-9 or HER ECD (when using cut-offs at 15 ng/mL for HER ECD and 0.12 μg/L for S100ß).

### Prognostic factors

**Fig. 1:**
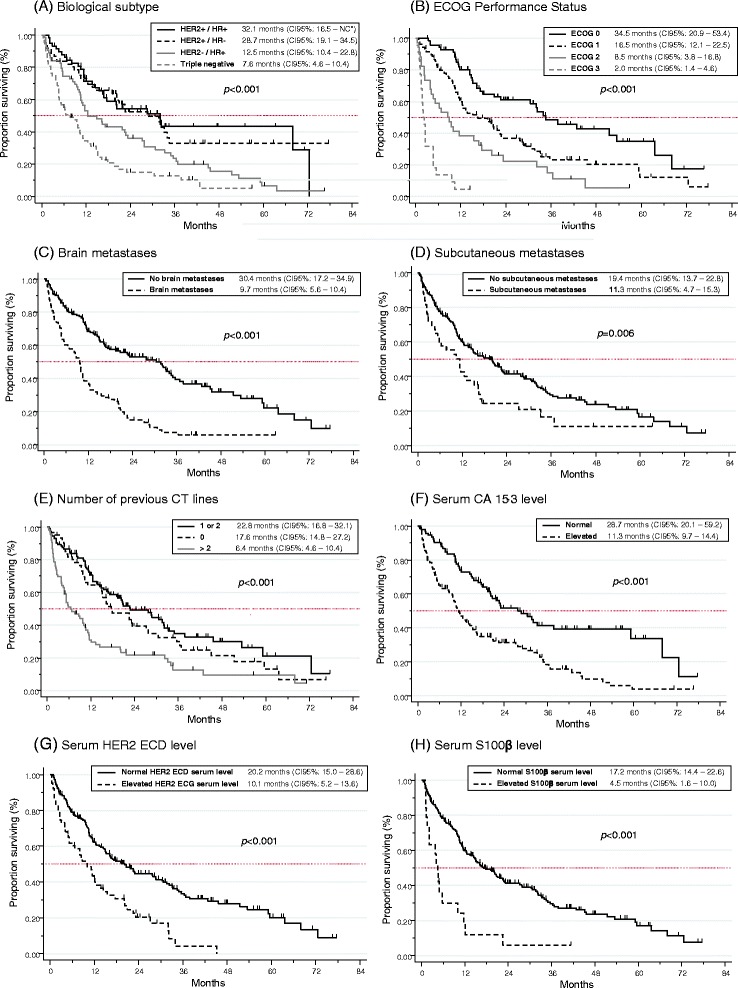
Overall survival (OS) according to **a** the tumor biological subtype, **b** the ECOG performance status, **c** the presence of brain metastases, **d** the presence of subcutaneous metastases, **e** the number of previous chemotherapy lines, **f** the serum CA 15-3 level, **g** the serum HER2 ECD level, and **h** the serum S100ß level. *NC: not calculable

**Fig. 2:**
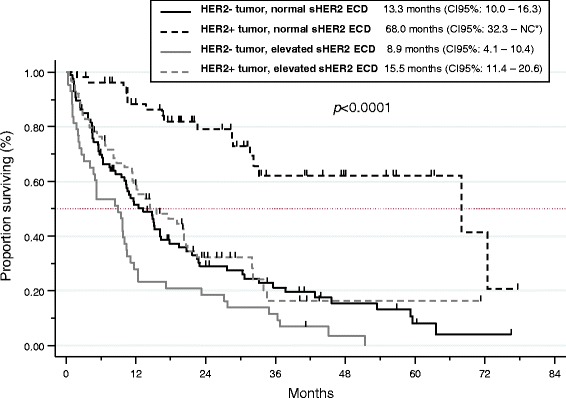
Overall survival (OS) according to the serum HER2 ECD level (with cut-off 15 ng/mL) in the HER-positive and HER2-negative populations. *NC: not calculable

At the time of the analysis, 69.2 % of patients had died, mostly because of MBC progression (87.9 %). With a median follow-up of 40.8 months, median OS was 16.2 months (95 % CI 12.4–20.6). The 1-year and 2-year survival rates were 57.1 % (95 % CI 50.6–63.1) and 38.5 % (95 % CI 32.1–44.9), respectively. The following variables were significant poor prognostic determinants in univariate analysis (Table 2): grade 3 tumors (*p* = 0.02), PR-negative tumors (*p* = 0.03), ER-negative tumors (trend, *p* = 0.09), HER2-negative tumors (*p* < 0.001), metachronous metastatic disease (*p* = 0.01), existence of two or more metastatic sites (*p* < 0.001), visceral metastases (compared with bone and/or subcutaneous metastases only) (*p* = 0.001), liver metastases (*p* < 0.001), BM (*p* < 0.001), subcutaneous metastases (*p* = 0.006), metastases from other sites (*p* < 0.001), number of previous metastatic CT lines >2 (*p* < 0.001), no previous anti-HER2 treatment (*p* < 0.001), poor ECOG performance status (*p* < 0.001), and low albumin levels (*p* < 0.001) (Fig. 1). Among the tested biomarkers, high CEA (*p* = 0.001), high CA 15-3 (*p* < 0.001), high NSE (*p* < 0.001), high HER2 ECD (*p* < 0.001 with cut-offs at 15 ng/mL and 30 ng/mL), high S100ß (*p* < 0.001 with cut-offs at 0.12 μg/L and 0.072 μg/L) and high MMP-9 (*p* = 0.009) serum levels were also poor prognostic determinants in univariate analysis (Fig. 1). The prognostic value of serum HER2 ECD (with cut-offs 15 and 30 ng/mL) was significant in patients with HER2-positive tumors as well as in the HER2-negative tumors group (*p* < 0.0001 and *p* = 0.005, respectively, with cut-off 15 ng/mL) (Fig. 2). A multivariate analysis was performed (*n* = 208), excluding albumin due to a high number of missing data. The ECOG performance status, the presence of BM or subcutaneous metastases, the number of previous metastatic CT lines, the tumor biological subtype, and high serum CA 15-3, S100ß (cut-off at 0.12 μg/L) and HER2 ECD (cut-off at 30 ng/mL) levels were independently associated with poor prognosis (Table 3). We performed another analysis considering the biomarkers as continuous variables and confirmed that high serum CEA (*p* < 0.001), CA15-3 (*p* = 0.003), NSE (*p* < 0.001), HER2 ECD (*p* = 0.009) and S100ß (*p* < 0.000), but not MMP-9 (*p* = 0.476), were significantly associated with survival in univariate analysis. In multivariate analysis, HER2 ECD and S100ß, but not CA 15-3, remained independent prognostic factors (*p* = 0.004 and *p* < 0.001, respectively).

**Table 2:** Univariate analysis

Parameter	Median OS in months (CI 95 %)	*P*-value
Initial characteristics		
* Tumor biology group*		<0.001
HER2+ HR+	32.1 (16.5 – NC)	
HER2+ HR-	28.7 (19.1–34.5)	
HER2- HR+	12.5 (10.4–22.8)	
Triple negative	7.6 (4.6–10.4)	
* ER status*		0.087
Negative	13.3 (10.0–19.1)	
Positive	20.1 (12.4–20.6)	
* PR status*		0.035
Negative	12.5 (10.6–17.8)	
Positive	22.8 (16.5–35.6)	
* HER2 status*		<0.001
Negative	10.4 (8.5–12.5)	
Positive	31.6 (20.2–34.5)	
* Histological subtype*		0.344
Ductal carcinoma	15.2 (11.6–20.6)	
All other subtypes	20.1 (10.1–35.6)	
* Histological grade (SBR)*		0.024
1 or 2	20.6 (14.8–31.6)	
3	11.1 (9.7–15.5)	
* Inflammatory BC*		0.523
No	15.1 (11.6–20.2)	
Yes	20.9 (6.8 – NC)	
* Metastatic status at BC diagnosis*		0.012
M0	13.6 (10.4–16.5)	
M1	27.2 (13.7–32.1)	
Characteristics at the time of the serum sample		
* Age group*		0.723
< 50	15.0 (10.0–23.0)	
50 to 70	16.3 (11.6–20.7)	
> 70	20.2 (9.7–51.4)	
* ECOG status*		<0.001
Score 0	34.5 (20.9–53.4)	
Score 1	16.5 (12.1–22.5)	
Score 2	8.5 (3.8–16.8)	
Score 3	2.0 (1.4–4.6)	
* Number of lines of CT*		<0.001
0 line	17.6 (14.8–27.2)	
1 or 2 line(s)	22.8 (16.8–32.1)	
> 2 lines	6.4 (4.6–10.4)	
* Previous anti-HER2 treatment*		<0.001
No	11.7 (9.8–15.2)	
Yes	28.7 (20.1–33.9)	
* Metastatic-free interval*		0.464
≤ 24 months	14.8 (10.6–20.7)	
> 24 months	16.5 (12.4–23.3)	
* Number of metastatic sites*		<0.001
1	59.2 (23.0 – NC)	
> 2	13.3 (11.1–16.8)	
* Location of metastatic sites*		0.001
Bone and/or subcutaneous only	63.6 (95 % CI NC)	
Visceral	14.4 (11.4–19.4)	
* Brain metastases*		<0.001
Absent	30.4 (17.2–34.9)	
Present	9.7 (5.6–10.4)	
* Liver metastases*		<0.001
Absent	28.7 (14.9–36.8)	
Present	12.4 (10.6–17.6)	
* Bone metastases*		0.639
Absent	17.8 (10.6–31.6)	
Present	15.2 (12.1–20.2)	
* Lung metastases*		0.096
Absent	20.7 (14.4–30.4)	
Present	12.4 (10.1–17.8)	
* Lymph node metastases*		0.296
Absent	19.4 (12.4–27.7)	
Present	13.6 (10.5–19.1)	
* Subcutaneous metastases*		0.006
Absent	19.4 (13.7–22.8)	
Present	11.3 (4.7–15.2)	
* Pleural metastases*		0.077
Absent	16.3 (12.5–20.9)	
Present	13.3 (5.2–28.7)	
* Metastases of other sites*		<0.001
Absent	20.9 (16.2–30.7)	
Present	11.1 (7.5–13.6)	
* Anemia*		0.134
No	15.5 (8.1–22.8)	
Yes	10.3 (8.1–11.7)	
* Leucopenia*		0.363
No	10.3 (8.5–12.4)	
Yes	10.8 (6.4–31.6)	
* Neutropenia*		0.908
No	10.6 (8.5–12.4)	
Yes	10.4 (5.2 – NC)	
* Lymphopenia*		0.351
No	11.1 (9.4–15.5)	
Yes	8.4 (4.7–10.6)	
* Thrombopenia*		0.748
No	10.6 (8.5–13.6)	
Yes	10.1 (2.6–20.6)	
* Serum protein level*		0.780
Normal	9.7 (5.2–11.6)	
Low	6.4 (2.3–13.6)	
* Serum albumin level*		<0.001
Normal	14.4 (11.1–19.4)	
Low	1.6 (1.1–2.4)	
* Serum LDH*		0.241
Normal	10.3 (5.5–13.7)	
Elevated	4.7 (1.9–9.8)	
* Elevated CEA*		0.001
Normal	20.6 (16.2–28.7)	
Elevated	10.6 (8.7–12.5)	
* Elevated CA 15-3*		<0.001
Normal	28.7 (20.1–59.2)	
Elevated	11.3 (9.7–14.4)	
* Serum HER2 (cut-off 15 ng/mL)*		<0.001
Normal	22.6 (16.2–32.3)	
Elevated	11.3 (8.9–14.4)	
* Serum HER2 (cut-off 30 ng/mL)*		<0.001
Normal	20.2 (15.0–28.6)	
Elevated	10.1 (5.2–13.6)	
* Serum NSE (cut-off 12.5 μg/L)*		<0.001
Normal	20.6 (16.2–30.4)	
Elevated	9.9 (5.6–14.4)	
* Serum MMP9 (cut-off 50 ng/mL)*		0.009
Normal	3.8 (1.1 – NC)	
Elevated	16.2 (12.5–20.7)	
* Serum S100ß (cut-off 0.12 μg/L)*		<0.001
Normal	17.2 (14.4–22.6)	
Elevated	4.5 (1.6–10.0)	
* Serum S100ß (cut-off 0.072 μg/L)*		<0.001
Normal	20.3 (15.1–30.4)	
Elevated	6.2 (4.5–12.1)	

*Abbreviations*: *ER* estrogen-receptors, *PR* progesterone-receptors, *SB* Scarf, Bloom and Richardson, *BC* breast cancer, *CT* chemotherapy, *LDH* Lactate Deshydrogenase, *CEA* Carcinoembryonic Antigen, *CA 15-3* Cancer Antigen 15-3, *HER2-ECD* HER2-extra-cellular domain, *NSE* Neuron Specific Enolase, *MMP-9* Matrix Metalloproteinase 9

**Table 3:** Multivariate Cox regression analyses (Stepwise procedure)

Parameter	Hazard-ratio	95 % CI	*P*-value
*Performance status:*			
ECOG 0	1		
ECOG 1	1.75	1.12–2.73	0.013
ECOG 2	2.62	1.51–4.53	0.001
ECOG 3	9.78	4.85–19.73	<0.001
*Tumor biology:*			
HER2+/HR+	1		
HER2+/HR−	1.19	0.70–2.04	0.514
HER2−/HR+	2.49	1.49–4.18	0.001
Triple negative	5.31	2.97–9.47	<0.001
*Number of previous metastatic CT lines:*			
0	1		
1 or 2	1.81	1.14–2.86	0.012
> 2	2.57	1.57–4.21	<0.001
*Brain metastases*	2.26	1.57–3.25	<0.001
*Subcutaneous metastases*	1.92	1.22–3.01	0.005
*Elevated CA 15-3 (cut-off 30 UI/L)*	1.95	1.31–2.93	0.001
*Elevated HER2 ECD (cut-off 30 ng/mL)*	2.51	1.53–4.12	<0.001
*Elevated S100ß (cut-off 0.12 μg/L)*	1.93	1.05–3.54	0.033

*Abbreviations*: *ECOG* Eastern Cooperative Oncology Group, *CT* chemotherapy, *CA 15-3* Cancer Antigen 15-3, *HER2-ECD* HER2-extra-cellular domain

## Discussion

### Prognostic value of serum CA15-3, HER2 ECD and S100ß

Our study confirmed the prognostic value of serum CA 15-3 [14–16]. On the contrary, serum CEA, that has previously been associated with worse outcome (progression-free survival and OS) in several studies, was correlated with poor prognosis in univariate but not multivariate analysis. This result is possibly linked to a preponderant effect of other more robust prognostic factors [14–16].

In addition to the prognostic value of CA 15-3, our study showed that high serum HER2 ECD and S100ß levels were independent unfavorable prognostic factors in MBC patients. The prognostic value of serum HER2 ECD appeared significant not only in HER2-positive tumors but also in HER2-negative tumors (*p* = 0.03). Discordance between the tumor HER2 status and the HER2 level have been previously reported [36]. In our study, 44 patients (33.6 %) with a HER2-negative tumor had an elevated serum HER2 ECD (≥15 ng/mL). The prognostic impact of serum HER2 ECD has already widely been studied in patients with HER2-positive MBC [17, 18]. However, it has been less frequently investigated in patients with HER2-negative MBC, with only few studies suggesting a prognostic value in this population as well [19]. Our result could have different explanations. First, it could be linked to phenotypic MBC heterogeneity, some tumor cells displaying HER2 overexpression among HER2-negative MBC [37]. It could also be due to changes in the HER2 status along tumor progression. However this phenomenon seems to be a rare event [38, 39]. In a recent study, discrepancies between the primary tumor and distant metastases as regards to the HER2 status were observed in only 8 % of cases [40]. Moreover, we collected the most recent tumor biology data, as some phenotypic changes are possible between the initial tumor and metastatic recurrences. Finally, it has been recently shown that some HER2-negative tumors (tumors that do not overexpress the HER2 ECD) are positive for the intracellular domain of HER2 and/or show an amplification of the HER2 gene [41]. In the study published by Panis et al., this group of patients (16 % of the patients with a HER2-negative tumor) had a survival similar to that of HER2-positive tumor patients. Moreover, the PI3K expression and the AKT pathway activation were similar to that of HER2-positive tumors. This phenomenon could be explained by an increase in the ECD cleaving, leading at the same time to an increase of serum ECD and of a negative HER2 tumor phenotype. We can thus hypothesize that the prognostic value of serum HER2 ECD in HER2-negative tumors could be linked in part to the subset of patients with “false” HER2-negativity, that is, patients without HER2 ECD expression but with expression of its intracellular, activated, domain.

The prognostic value of S100ß in MBC has been scarcely investigated in breast cancer so far, while it has been well demonstrated in melanoma [29]. We found high serum S100ß to be an independent unfavorable prognostic factor. This is concordant with the results of another study on a series of 80 EBC and MBC patients [42]. In this study, elevated serum S100ß (cut-off 0.12 μg/L) was a significant predictor of poor disease-free survival. However, our result must be considered with caution as the two groups identified by S100ß serum levels were grossly unbalanced regarding the number of patients (*n* = 19 for S100ß > 0.12 μg/L compared to *n* = 224 for normal S100ß). Despite its significant prognostic value in multivariate analysis, its clinical impact is questionable, as rarely elevated biomarkers appear to be of limited usefulness in clinical practice. It is interesting to note that the analysis of S100ß with the cut-off of 0.072 μg/L, even though it defined two more balanced groups regarding the number of patients, did not reach significance in multivariate analysis. Our study has included a high number of patients with BM (35 %, as discussed in the “limitations” section), and BM are associated with a poor outcome. Among these patients, 73 (82.9 %) had a S100ß serum level > 0.12 μg/L. It could thus be hypothesized that the negative prognostic impact of high serum S100ß is linked to the presence of BM. However, the serum S100ß was not predictive of BM in a series of 101 patients with BC [33]. This question is also being studied in another article from our team (under review).

We found that neither NSE nor MMP-9 serum levels were correlated with outcomes. The prognostic value of serum NSE in MBC patients had, to our knowledge, never been studied before. Despite its prognostic value in univariate analysis, we found that it was not an independent prognostic factor in this population.

High serum MMP-9 has been associated with poor disease-free survival and OS in previous studies in breast cancer patients [25, 35]. We found a reverse association in univariate analysis: patients with high serum MMP-9 had a better outcome in our study. However, MMP-9 did not remain an independent prognostic factor in multivariate analysis. Several hypotheses can be made to explain this discordant result. First, it could be due to differences in the population’s initial characteristics, as the study by Sung et al. evaluated MMP-9 tissue expression in EBC, while our study included advanced MBC patients [25]. One can thus hypothesize a differential effect between the role of MMP-9 in invasion (linked to EBC relapse) and its lack of prognostic impact in later stage MBC, for whom the invasion and metastatic process has already been set. Another explanation could be linked to the different method of MMP-9 evaluation, based in one of the previous studies on tissue expression, and on serum levels in our present study. Other limitation for the analysis of the prognostic impact of MMP-9 on outcomes is the absence of a validated cut-off. We used a previously reported cut-off of 50 ng/mL, but other cut-offs may be more pertinent [35]. Finally, the two groups defined by MMP-9 serum levels in our study were very unbalanced (*n* = 6 for MMP-9 ≤ 50 ng/mL compared with *n* = 244 for MMP-9 > 50 ng/mL), which may explain our negative results. Serum MMP-9 being elevated in a vast majority of patients, its clinical usefulness is questionable.

### Prognostic value of other biological parameters

The classical prognostic value of HR status was confirmed in our study (median OS 22.8 months for PR-positive tumors compared with 12.5 months for PR-negative tumors; *p* = 0.035) [4, 7, 8]. Concerning the ER status, this difference in OS did not reach significance, which can be explained by the fact that PR positivity ensures a functionally intact estrogen response pathway in ER-positive tumors, and appears in different studies as a strong indicator of ER sensitivity.

Regarding the tumor HER2 status, we found that the presence of HER2 hyper-expression/amplification was an independent favorable prognostic factor in MBC. Median OS was 31.6 months in the HER2-positive group compared with 10.4 months in the HER2-negative group (*p* < 0.001). This result is concordant with that of other recent studies (considering the 2008–2015 inclusion period of our study) conducted in the Trastuzumab field, but discordant with pre-Trastuzumab studies [9, 10]. Indeed, in the study published by Dawood et al., the outcome was more favorable in patients with HER2-positive tumors treated with Trastuzumab than in patients with HER2-positive tumors not receiving Trastuzumab, or than in patients with HER2-negative tumors [10]. In our study, patients who had received a previous anti-HER2 treatment had, indeed, a better outcome (median OS 28.7 months compared with 11.7 months for patients not receiving anti-HER2 treatments, *p* < 0.001).

We found significant differences in median OS between the four biological groups of tumors, confirming previous data. Patients with HER2+/HR+ tumors have the best outcome, with a median OS of 32.1 months in our study, consistent with the 34.4 month median OS reported in a previous study [5]. Triple negative tumors have the most dismal outcome, with a median OS of 7.6 months in our study consistent with the 8.8 month median OS previously reported [5]. HER2+/HR- and HER2-/HR+ tumors present with an intermediate survival (median OS of 28.7 months and 12.5 months, respectively). In the study published by Lobbezoo et al., patients with HER2-/HR+ tumors had a better outcome than patients with HER2+/HR- tumors, conversely to what was found in our study [5]. This difference could have two explanations. First, only 27.0 % of patients with HER2-positive tumors received Trastuzumab in this study, compared 83.2 % in our study (and only 37.5 % versus 80.3 % of patients with HER+/HR- tumors, in the Lobbezoo et al. study and the present work, respectively). Moreover, a more heterogeneous population of patients was included in our study, with a variable number of previous CT lines (range 0–9), whereas Lobbezoo et al. have investigated prognostic factors at the time of the diagnosis of the metastatic disease.

Other primary tumor characteristics, such as the histological subtype or the histological grade, were not correlated with the patients’ prognosis. This is concordant with results from other large studies in MBC [5, 11, 43]. However the histological grade was shown to have a prognostic value in other studies [4, 44].

Serum LDH was not found to be predictive of the patients’ outcomes in both univariate and multivariate analysis, which is discordant with the results of other studies [11, 13]. The high proportion of missing data (*n* = 160) probably explains our result. Also due to a high number of missing data, the albumin serum level could not be included in the multivariate Cox model, however a high level had a negative prognostic value in univariate analysis, and has been reported as an independent prognostic factor of poor outcome in previous studies [12, 13].

### Clinical prognostic factors

Among clinical parameters associated with MBC patients’ outcome, the number of metastatic sites and the metastatic sites involved are both of high value. The presence of bone and/or subcutaneous metastases are associated with a better outcome (median OS >33 months) compared with the presence of multiple or visceral metastases (median OS 22 months) [6]. In our study, the occurrence of visceral metastases (compared with bone and/or subcutaneous metastases only) was a poor prognostic factor, however this results must be considered with caution as only few patients had isolated bone and/or subcutaneous metastases (*n* = 13), due to a high proportion of patients with advanced MBC. The presence of BM was associated with a poor outcome in our study (median OS of 9.7 months compared with 30.4 months, *p* < 0.001), as shown in previous studies [4]. However, the presence of liver metastases was associated with poor prognosis in univariate but not multivariate analyses, while its poor prognostic value has been demonstrated in previous studies [4]. This difference may be due to the over-representation of BM in our study. As shown in previous studies, a high number of metastatic sites was a poor prognostic determinant in univariate but not in multivariate analyses [7].

Surprisingly, we found that *de novo* MBC (compared to metachronous metastatic disease) was associated with a better outcome in univariate analysis. A trend favoring *de novo* MBC was also found in a study published by Lobbezoo et al., with a median OS of 21.1 months for patients with metachronous metastatic progression compared with 29.4 months for patients with *de novo* MBC (*p* = 0.14) [45]. In this study, the prognostic impact of the MFI was also investigated: *de novo* MBC patients had a better outcome than patients with a MFI < 24 months, but no difference in OS was reported when comparing *de novo* MBC patients with patients who had a MFI > 24 months, advocating for the identification of this short MFI group as a highly aggressive, resistant, tumor subset [44]. These results are somewhat discordant with numerous other studies that have shown that patients with a MFI < 24 months had a poorer outcome compared with patients with a MFI ≥ 24 months [4, 7, 8, 11]. However, in these studies, patients with a synchronous metastatic disease were classified into the MFI < 24 months group, possibly diluting the positive prognostic impact of synchronous metastases within the short MFI group. In our study, we found no difference in OS among patients according to the MFI (*p* = 0.464).

The number of previous metastatic CT lines was also an independent prognostic determinant in our study. Patients with no previous CT line had a better outcome compared to those having received one or two CT line(s), or >2 lines, which is concordant with previously published data [7].

### Limitations

Our study is based on a well-characterized series of MBC patients and brings new data on additional tumors markers and their prognostic value. Nevertheless, it presents some limitations. Due to its retrospective nature, we could not avoid having some missing data, in particular for classical biological parameters such as LDH or albumin. Moreover, patients in our study were identified for the purposes of a case-control study in MBC patients with BM, therefore there is in our series an over-representation of these patients, and, as a consequence, of patients with a HER2-positive tumor. Indeed, 35 % of patients had BM, compared to a few percent in other studies [4, 5]. This result causes an obvious bias regarding the survival analyses. The median OS in our study was indeed lower than in previous works: 16.2 months compared with 23.1 months and 21.8 months in the studies published by Largillier et al. and Lobbezoo et al., respectively [4, 5]. This difference can also be explained by the fact that patients in our series had a more advanced disease. Indeed, 30.4 % of patients had an advanced metastatic disease (>2 previous CT lines) while patients in the Lobbezoo et al. study were included at the diagnosis of the metastatic disease [5]. As regards the HER2 tumor status, a high proportion (47.6 %) of patients had a HER2-positive tumor, whereas this proportion is usually around 20 % in various studies [4, 5]. Therefore, our population may not have been entirely representative of a routine MBC population. However, the analysis on the 162 MBC patients without BM confirmed that high serum CA 15-3 (*p* < 0.001), HER2 ECD (*p* < 0.001) and S100ß (*p* < 0.001) levels were associated with poor survival.

## Conclusions

In conclusion, serum CA 15-3, HER2 ECD and S100ß levels could represent useful independent prognostic factors in MBC. A validation of these results in an independent set of MBC samples is necessary to confirm these findings. Of particular interest is the independent prognostic value of serum HER2 ECD level, independently of the tumor HER2 status, possibly linked to metastatic tumor heterogeneity or presence of HER2 ECD negative/HER2 intracellular activated tumors.

## Abbreviations

BM, brain metastases; CA 15-3, cancer antigen 15-2; CEA, carcinoembryonic antigen; CI, confidence interval; CISH, chromogenic in situ hybridization; CT, chemotherapy; EBC, early breast cancer; ECD, extra-cellular domain; ER, estrogen receptors; FISH, fluorescence in situ hybridization; HER2, human epidermal growth factor 2; HR, hormone-receptors; IHC, immunohistochemistry; LDH, lactate dehydrogenase; MBC, metastatic breast cancer; MFI, metastatic-free interval; MMP-9, matrix metalloproteinase 9; NSE, neuron specific enolase; OS, overall survival; PR, progesterone receptors
